# Occupational stress and psychological wellbeing among critical care nurses in private hospitals in Somalia: a national cross-sectional study

**DOI:** 10.3389/fpubh.2026.1932017

**Published:** 2026-08-25

**Authors:** Abdullahi Hassan Elmi, Rasha Kadri Ibrahim, Rayaan Abdirahman Hassan, Fahma Hussein Ibrahim

**Affiliations:** 1 Department of Nursing and Midwifery, Sumait Hospital, SIMAD University, Mogadishu, Somalia; 2 Faculty of Medicine and Health Sciences, SIMAD University, Mogadishu, Somalia; 3 Nursing Department, Fatima College of Health Science, Madinat Zayed, United Arab Emirates; 4 Faculty of Medicine and Health Sciences, Sumait Hospital, SIMAD University, Mogadishu, Somalia; 5 Department of Obstetrics and Gynecology, Sumait Hospitals, SIMAD University, Mogadishu, Somalia

**Keywords:** critical care nursing, intensive care units, nurses, occupational stress, psychological wellbeing, Somalia

## Abstract

**Background:**

Critical care nurses operate in some of the most demanding environments within the health system, and their psychological wellbeing is closely tied to patient outcomes and workforce sustainability. Somalia's fragile healthcare system, marked by persistent resource shortages and limited regulation, presents additional pressures that may elevate stress among ICU nurses. However, evidence from the Somali context has been lacking. This study examined the relationship between occupational stress and psychological wellbeing among critical care nurses working in private intensive care units across Somalia.

**Methods:**

A nationwide cross-sectional survey was conducted among all 134 eligible critical care nurses employed in the ICUs of 13 private hospitals. Data were collected using a structured online questionnaire that included demographic characteristics, the Perceived Stress Scale (PSS-10), and a modified version of Ryff's Psychological Wellbeing Scale. Descriptive statistics were used to summarize participant characteristics. Pearson correlation assessed the relationship between stress and wellbeing, and multiple linear regression identified predictors of psychological wellbeing.

**Results:**

Most participants reported moderate stress (64.9%), while nearly one-third experienced high stress. Overall psychological wellbeing was moderate, with the highest scores observed in purpose in life and positive relations. No significant correlation was found between perceived stress and psychological wellbeing (*r* = −0.077, *p* = 0.377). Regression analysis showed that female nurses had significantly lower psychological wellbeing (*p* = 0.001), while nurses with chronic illnesses reported higher wellbeing (*p* = 0.042). Other demographic and work-related variables showed no significant associations.

**Conclusion:**

Critical care nurses in Somalia reported moderate occupational stress alongside moderate psychological wellbeing, with no significant association observed between the two variables. Gender disparities and the unexpected association between chronic illness and psychological wellbeing highlight the complexity of psychological outcomes in this setting. Strengthening staffing, improving working conditions, and implementing targeted psychological support services may help support the wellbeing of this vital workforce.

## Introduction

The healthcare system in Somalia is characterized by resource constraints, a fragmented structure, and critical workforce shortages, all of which have been shaped by decades of instability (1–3). 20% of health services are provided by the public sector, mainly in urban areas. In the private sector, often unregulated, most of the care is provided, leaving rural and displaced populations with minimal access. Only 35% or less of the population has access to essential health services, and health indicators such as maternal and child mortality remain among the worst globally (4, 5).

The health workforce in Somalia is severely inadequate and unevenly distributed; there are only 0.4 to 4.5 doctors, nurses, and midwives for every 10,000 people, far below the WHO minimum requirement of 23 per 10,000 (1–3, 6). Additionally, the nursing workforce faces issues such as insufficient training, a lack of standardized qualifications, poor regulation, and lower retention rates due to inadequate pay and difficult working conditions (6, 7). This shortage of nurses and health workers is a significant barrier to achieving universal health coverage and meeting the population's essential care needs (1–3, 6).

Critical care nurses provide continuous care for critically ill patients in intensive care units, where they manage complex clinical conditions, respond to emergencies, and collaborate with multidisciplinary teams. The high-acuity nature of critical care, combined with heavy workloads, rapid decision-making, prolonged shifts, and frequent exposure to critically ill patients, places these nurses at increased risk of occupational stress and adverse psychological outcomes (8–13).

Critical care nurses consistently experience higher levels of occupational stress than nurses in many other clinical settings because of heavy workloads, high patient acuity, frequent emergencies, emotional demands, and organizational challenges. Studies from both high- and low-resource settings have reported moderate-to-high levels of occupational stress among critical care nurses, with resource limitations, workforce shortages, and organizational constraints further exacerbating stress in fragile health systems (14–22).

The Job Demands–Resources (JD-R) model provides a useful framework for understanding occupational stress among critical care nurses. The model proposes that high job demands, such as heavy workload, emotional strain, and resource limitations, increase occupational stress, whereas job resources, including organizational support, adequate staffing, and professional autonomy, help protect psychological wellbeing. This framework provides a conceptual basis for examining the relationship between occupational stress and psychological wellbeing among critical care nurses in the Somali healthcare context.

The World Health Organization identifies occupational stress as a global epidemic, affecting up to 90% of medical consultations and leading to absenteeism, employee turnover, and diminished quality of service (18). The healthcare sector has the highest estimated prevalence of work-related stress in the UK and across Europe (23). Mitigating workplace stress is crucial for attaining global health objectives and preserving workforce wellbeing. Critical care employment universally elevates occupational stress owing to its rigorous, high-pressure setting. Although the fundamental stressors are globally comparable, limitations in resources and organizational elements can exacerbate stress in developing nations. Global data underscore the pressing necessity for focused measures to safeguard the wellbeing of healthcare workers and the quality of patient care (20).

Occupational stress among critical care nurses is significantly correlated with negative psychological consequences, including burnout, anxiety, depression, and reduced job satisfaction (24, 25). In diverse healthcare settings, elevated occupational stress is consistently associated with increased occurrences of burnout, emotional fatigue, and symptoms of anxiety and depression among practitioners (26, 27). Burnout acts as a mediator between occupational stress and anxiety, increasing the risk of psychological suffering (28, 29). Factors such as excessive workload, insufficient organizational support, and exposure to patients in distress or confronting mortality markedly exacerbate these outcomes (27).

The psychological impact of professional stress on nurses directly affects patient care and the sustainability of healthcare systems. Stressed and emotionally fatigued employees are more likely to report diminished job satisfaction, increased absenteeism, and elevated turnover rates, all of which adversely affect the quality of patient care (25, 30). Burnout and compromised mental health among healthcare professionals are associated with a rise in medical errors, negative outcomes, and declining patient satisfaction. At the systemic level, these challenges jeopardize the sustainability of healthcare provision by depleting resources, escalating expenses, and destabilizing the workforce (31).

Considering abundant data from other nations, a considerable knowledge deficit exists concerning occupational stress and its psychological and systemic consequences among critical care nurses in Somalia. No research was found that directly examines these concerns within the Somali setting. The research gap underscores the pressing need for context-specific studies to guide initiatives and policy in Somalia. This study aimed to examine the relationship between occupational stress and psychological wellbeing among critical care nurses working in private intensive care units in Somalia and to identify demographic and work-related predictors of psychological wellbeing.

## Methods

### Study design and setting

This study adopted a nationwide descriptive cross-sectional approach to explore the Relationship Between Occupational Stress and Psychological Wellbeing Among Critical Care Nurses in Somalia. All intensive care units (ICUs) operating within the country's private healthcare system were included. In total, 13 private hospitals with functioning ICUs participated, providing a complete picture of the Occupational Stress and Psychological Wellbeing encountered by Somali CCNs.

### Subjects

The study aimed to include the entire population of 134 nurses employed across the ICUs of the 13 participating private hospitals. Eligibility was limited to registered nurses who had worked in their current ICU for a minimum of 6 months, ensuring adequate exposure to the critical care environment. Nurses on prolonged leave or those with less than 6 months of ICU experience were excluded to maintain uniformity in the sample and ensure reliable assessment of occupational stress and psychological wellbeing.

### Sample size determination

Given that this study aimed to include all critical care nurses (CCNs) working in Somalia's private ICUs, a census approach was adopted instead of traditional sampling. All 134 eligible nurses were invited to take part, removing the necessity for a formal sample size calculation. This complete inclusion strengthened both the representativeness and the reliability of the study's findings.

### Data collection

Data were collected using a structured electronic questionnaire administered via Google Forms. The survey link was distributed to all eligible critical care nurses through the nursing managers of the participating ICUs in the 13 private hospitals. Before distribution, the investigators coordinated with each hospital to identify all eligible nurses based on the predefined inclusion criteria. Nursing managers facilitated communication with eligible participants and issued follow-up reminders during the data collection period to maximize participation. Questionnaire completion was monitored relative to the number of eligible nurses at each hospital while maintaining participant anonymity. Data collection continued until responses were received from all 134 eligible nurses, resulting in a 100% response rate.

## Instruments

### Characteristics of nurses

The demographic section captured details including participants' age, gender, marital status, educational qualification, income level, presence of chronic illness, nurse-to-patient ratio, shift duration, overall nursing experience, and critical care experience.

### Perceived Stress Scale (PSS)

The PSS was originally developed by Cohen, Kamarck, and Mermelstein (1983) (32). It comprises 10 items rated on a 5-point Likert scale ranging from 0 (never) to 4 (very often). Certain items (4, 5, 6, 7, 9, and 10) are reverse-scored to maintain scale balance. Total scores range from 0 to 40, with higher scores indicating greater perceived stress. Stress levels are classified as low (0–13), moderate (14–26), or high (27–40). In this study, the PSS demonstrated robust internal consistency, with a Cronbach's alpha of 0.81, confirming its reliability for assessing stress among critical care nurses in Somali ICUs (33). For the PSS, participants' responses to reverse-scored items were recalculated (0 → 4, 1 → 3, 2 → 2, 3 → 1, 4 → 0), and total scores were computed by summing all items. For psychological wellbeing, reverse-scored items were recalibrated using the formula: [(Number of scale points) + 1]-(Respondent's answer). Subscale totals were derived by summing relevant items for each domain, with higher scores reflecting better wellbeing.

### Psychological wellbeing

Psychological wellbeing was assessed using Ryff's Psychological Wellbeing Scale (34). This section included 18 items, each with three facets across six dimensions: autonomy, environmental mastery, personal growth, positive relations with others, purpose in life, and self-acceptance. Responses were rated on a 7-point Likert scale from “Strongly Agree” to “Strongly Disagree,” with several items reverse-scored according to standard procedures. The Ryff Psychological WellBeing Scale also demonstrated good internal consistency in the current study, with a Cronbach's alpha coefficient of 0.88, supporting the reliability of the instrument. Higher scores indicated greater levels of psychological wellbeing. Subscale scores were calculated by summing item responses for each dimension, ensuring a precise evaluation of participants' psychological functioning.

### Validity and reliability

The questionnaire underwent content validation by a panel of nine seasoned Somali healthcare professionals, including three senior nurse educators, four nurse managers, and two hospital quality officers, each with over 8 years of professional experience. The panel assessed the instrument for clarity, relevance, and cultural appropriateness. Feedback was gathered through structured review forms and follow-up discussions, leading to minor adjustments that enhanced the questionnaire's precision and contextual suitability. This validation approach can be applied in similar research by engaging experts with equivalent experience and employing a structured review process (35). To further ensure the tool's cultural relevance and functionality, a pilot study was conducted with 20 nurses. The instrument demonstrated high internal consistency, as evidenced by Cronbach's alpha (33).

### Statistical analysis of the data

The data were analyzed using SPSS 27. Range (minimum and maximum), mean, and standard deviation were used. Categorical data were summarized as numbers and percentages. Pearson's correlation coefficient was used to assess the relationship between the variables in the study. Multiple linear regression analysis was performed to identify predictors of psychological wellbeing. Prior to model fitting, the assumptions of multiple linear regression were evaluated. Normality of residuals was assessed using the Kolmogorov–Smirnov test, and multicollinearity among the independent variables was examined. The diagnostic assessment indicated that these assumptions were adequately satisfied, supporting the use of multiple linear regression. The independent variables in the multiple regression models were overall perceived stress and sociodemographic data, and the dependent variable was psychological wellbeing.

## Results

Table 1 The response rate was 100% after contacting all participants. Slightly more than three-quarters of participants were male (76.1%), with 53% married. The largest percentages of nurses fall within the age categories 25–30 years and >30 years, each comprising 40.3% of the population. The majority reported no chronic disease (80.6%). In terms of work shifts, nurses work different lengths, with 8-h shifts being the most common (43.3%). Concerning experience levels, around 44.8% of nurses possess 4–6 years of overall nursing experience. A comparable trend is noted in critical care experience, with 44.8% having worked 4–6 years in this field.

**Table 1:** Distribution of the critical care nurses in somalia according to demographic (*n* = 134).

Socio-demographic characteristics	no.	%
Gender		
Male	102	76.1
Female	32	23.9
Age		
< 25	26	19.4
25–30	54	40.3
>30	54	40.3
Marital status		
Single	59	44.0
Married	71	53.0
Widow	4	3.0
Educational qualification		
Bachelor	88	65.7
Master	46	34.3
PhD	0	0.0
Income level		
< 300	31	23.1
301–500	55	41.0
>500	48	35.8
Have chronic illness		
Yes	26	19.4
No	108	80.6
Nurse patient ratio		
1:2 or less	80	59.7
1:3	29	21.6
More than 1:3	25	18.7
Shifting		
8 h	58	43.3
12h	36	26.9
16h	40	29.9
Overall nursing experience		
1–3 years	31	23.1
4–6 years	60	44.8
≥7 years	43	32.1
Critical care experience		
1–3 years	32	23.9
4–6 years	60	44.8
≥7 years	42	31.3

The findings in Table 2 reveal notable variations across the six domains of psychological wellbeing among Somali critical care nurses (*n* = 134). Overall psychological wellbeing was moderate (46.10%), with a mean score of 67.79 ± 8.84. In terms of subscales, the “purpose of life” subscale had the highest mean score, 55.31% (12.96 ± 2.51), followed by the positive relations subscale, 53.61% (12.65 ± 2.78). The self-acceptance subscale had the lowest, at 38.85% (9.99 ± 2.71).

**Table 2:** Distribution of Somalian critical care nurses according to mean scores of psychological wellbeing **(***n* = 134).

Study variables	Mean	SD	Min	Max	Mean percent	Rank
The autonomy	10.49	3.06	3.0	21.0	41.58	**5**
The environmental mastery	11.10	3.18	3.0	20.0	44.98	**3**
The personal growth	10.61	3.27	3.0	19.0	42.29	**4**
The positive relations	12.65	2.78	3.0	19.0	53.61	**2**
The purpose in life	12.96	2.51	6.0	20.0	55.31	**1**
The self-acceptance	9.99	2.71	3.0	19.0	38.85	**6**
**Total (18-126)**	**67.79**	**8.84**	**31.0**	**89.0**	**46.10**	

SD, Standard deviation.

Table 3 shows that the overall perceived stress was moderate (57.63 %), with a mean score of 23.05 ± 6.21. 64.9% of nurses experienced a moderate level of stress. 29.9% of participants reported experiencing a high level of stress.

**Table 3:** Distribution of Somalian critical care nurses according to mean scores of perceived stress (*n* = 134).

	No.	%
Level of perceived stress scale		
Low (0–13)	7	5.2
Moderate (14–26)	87	64.9
High (27–40)	40	29.9
Total score (0–40)		
Min.–Max.	4.0–36.0	
Mean ± SD.	23.05 ± 6.21	
**Percent score (Mean** **±SD)**	57.63 ± 15.52	

Regarding the correlation analysis in Table 4, a negative, non-significant correlation was found between the perceived stress scale and the overall psychological wellbeing of critical care Somali nurses, *r*(132) = −0.077, *p* = 0.377.

**Table 4:** Correlation between the variables of the study (*n* = 134).

	*r*	*p*
Perceived stress scale vs. psychological wellbeing	−0.077	0.377

*r*, Pearson coefficient.

Table 5 Female nurses showed considerably worse psychological wellbeing scores than men (*B* = −6.797, *p* = 0.001). This suggests that after controlling for all other variables, being female is associated with a 6.8-point decline in psychological wellbeing. Nurses with a chronic illness had substantially higher psychological wellbeing scores (*B* = 5.328; *p* = 0.042). Although unexpected, this may indicate stronger coping strategies, greater resilience, or more robust support mechanisms among nurses managing chronic diseases.

**Table 5:** Multivariate linear regression analysis for the parameters affecting psychological wellbeing in total sample (*n* = 134).

	*B*	SE	*T*	*p*	LL–UL 95% CI
Gender					
Male	0.000				
Female	−6.797	2.047	3.320	0.001^*^	−10.852 to −2.742
Age					
< 25	0.000				
25–30	−1.827	2.389	0.765	0.446	−6.558 to 2.905
>30	−2.253	2.860	0.788	0.432	−7.917 to 3.410
Marital status					
Single	0.000				
Married	1.933	1.853	1.043	0.299	−1.738 to 5.604
Widow	4.302	4.559	0.944	0.347	−4.726 to 13.331
Educational qualification					
Bachelor	0.000				
Master	1.589	2.008	0.791	0.430	−2.389 to 5.566
PhD	–	–	–	–	–
Income level					
< 300	0.000				
301–500	2.050	2.135	0.960	0.339	−2.178 to 6.278
>500	0.756	2.190	0.345	0.731	−3.581 to 5.093
Have chronic illness					
Yes	5.328	2.591	2.056	0.042^*^	0.196 to 10.460
No	0.000				
Nurse patient ratio					
1:2 or less	0.000				
1:3	3.180	2.128	1.494	0.138	−1.036 to 7.395
More than 1:3	−2.723	2.283	1.193	0.235	−7.244 to 1.798
Shifting					
8 h	0.000				
12 h	−2.030	1.936	1.048	0.297	−5.864 to 1.805
16 h	−0.172	1.905	0.090	0.928	−3.944 to 3.601
Overall nursing experience					
1–3 years	0.000				
4–6 years	0.223	2.928	0.076	0.939	−5.575 to 6.022
≥7 years	0.400	3.141	0.127	0.899	−6.620 to 5.820
Critical care experience					
1–3 years	0.000				
4–6 years	−0.004	3.016	0.001	0.999	−5.979 to 5.970
≥7 years	2.387	3.346	0.713	0.477	−4.240 to 9.013

**Model fit:** *R*^2^ = 0.296; Adjusted *R*^2^ = 0.186; *F* = 2.684; *p* = 0.001. B, Unstandardized Coefficients; SE, Standard error; CI, Confidence interval; LL, Lower Limit; UL, Upper Limit. ^*^, Statistically significant at *p* ≤ 0.05.

All other demographic and work-related variables (age, marital status, education, income level, nurse-patient ratio, shift duration, and experience) had no significant impact on psychological wellbeing (*p* > 0.05). Their 95% confidence intervals likewise crossed zero, indicating that the amended model had no significant predictive value.

## Discussion

This study examined the relationship between occupational stress and psychological wellbeing among critical care nurses working in private intensive care units across Somalia and identified demographic and work-related predictors of psychological wellbeing. The findings showed that although most nurses experienced moderate to high levels of occupational stress, no significant association was observed between occupational stress and psychological wellbeing. These findings reflect the wider pressures inherent in Somalia's fragile healthcare system, where chronic workforce shortages, resource limitations, and fragmented governance persist.

The moderate to high levels of perceived stress found in this study reflect the wider pressures inherent in fragile health systems where chronic workforce shortages, resource limitations, and fragmented governance persist (1–6). Similar stress patterns have been documented in Ethiopia, Ghana, Vietnam, China, and Brazil, where nurses in high-acuity units frequently report moderate-to-severe occupational stress attributed to workload demands, inadequate staffing, and organizational constraints (14–17). Given Somalia's ongoing recovery from decades of instability and the scarcity of health professionals, which is estimated to be far below WHO recommendations (3, 6). These findings are consistent with global evidence showing that critical care environments intensify stress in settings with limited health system capacity (21, 22).

The relatively preserved levels of psychological wellbeing suggest that critical care nurses may derive meaning and resilience from their professional roles despite working under demanding conditions. These findings align with research suggesting that nurses in high-intensity clinical environments often derive meaning, purpose, and professional identity from their roles, especially in contexts where patient acuity is high, and teamwork is essential (8–10). Positive interpersonal relationships may also reflect the collaborative nature of ICU practice, where coordinated decision-making and interdependence are central to patient survival (11, 12). The lower scores in self-acceptance echo previous studies showing that the emotional demands of critical care nursing, combined with exposure to trauma, rapid deterioration, and frequent encounters with mortality, can compromise self-esteem and personal confidence (24, 25). Emotional strain and burnout have long been associated with reduced self-acceptance and self-efficacy among healthcare professionals (27, 36).

The principal finding of this study was the absence of a statistically significant relationship between occupational stress and psychological wellbeing among critical care nurses in private intensive care units in Somalia. This finding contrasts with much of the existing literature, which has consistently reported an inverse association between occupational stress and adverse psychological outcomes, including reduced psychological wellbeing, burnout, anxiety, and depression among healthcare professionals (24–29). Several contextual and methodological factors may explain this discrepancy. Critical care nurses across the participating hospitals worked in broadly similar environments marked by persistent workforce shortages, limited resources, and demanding clinical responsibilities, which may have reduced variability in both occupational stress and psychological wellbeing. In addition, the cross-sectional design captured stress and psychological wellbeing at a single point in time, whereas the psychological consequences of occupational stress may evolve gradually and may not be fully reflected in concurrent measurements. Cultural factors may also have influenced participants' perceptions and reporting of psychological wellbeing, as resilience, communal support, and social expectations surrounding emotional expression may shape how stress is experienced and reported within the Somali context. Furthermore, although standardized and validated instruments were used, they may not fully capture culturally specific dimensions of psychological wellbeing. Therefore, the absence of a statistically significant association should not be interpreted as evidence that occupational stress has no influence on psychological wellbeing. Rather, it highlights the complexity of this relationship within Somalia's healthcare context and underscores the need for longitudinal and mixed-methods studies to better understand the mechanisms linking occupational stress and psychological wellbeing.

The observed gender differences may reflect the greater emotional and social burdens experienced by female nurses in high-intensity clinical settings. This finding mirrors evidence from several global studies showing that female healthcare workers are more likely to experience emotional exhaustion, heavier domestic responsibilities, and disproportionate psychological burdens in high-intensity workplaces (25, 37, 38). Social expectations within Somali society may further compound these pressures, intensifying the observed gender disparity.

Unexpectedly, nurses with chronic illnesses demonstrated higher psychological wellbeing. This finding differs from the pattern reported in most previous studies. One possible explanation is that living with a chronic condition may encourage the development of adaptive coping strategies or greater use of available social support. However, this interpretation remains speculative and should be interpreted with caution. Alternative explanations, including response bias, the healthy worker effect, reverse causality, or the influence of unmeasured confounding factors, cannot be excluded given the cross-sectional design. Future longitudinal and qualitative studies are needed to clarify the mechanisms underlying this association.

Other demographic and occupational variables, including age, marital status, education, income level, patient ratios, shift length, and years of experience, were not significant predictors of psychological wellbeing. In contrast, studies in Ethiopia, Jordan, Iran, and other regions have reported strong associations between workplace factors and psychological health (14, 20, 39–41). One possible explanation for the absence of similar associations in the present study is that the widespread resource limitations and organizational challenges characterizing Somalia's healthcare system may reduce variability in nurses' work experiences. However, this interpretation should be viewed cautiously and warrants further investigation in similar low-resource settings.

These findings have important implications for workforce development, policy, and nursing practice. The persistent levels of occupational stress highlight the urgent need to strengthen ICU staffing capacity, promote stable nurse-to-patient ratios, and ensure adequate organizational support. Psychological wellbeing programs, including counseling services, stress-management training, and peer-support initiatives, could help enhance nurse resilience and reduce burnout. Given the gender-related disparities, interventions should also be designed with attention to the specific challenges that female nurses encounter. Strengthening governance, establishing regulatory standards for private-sector ICU operations, and improving job security may further enhance both nurse wellbeing and patient safety (12, 21, 40).

Although the study's findings provide valuable national insights, several limitations should be considered. The cross-sectional design prevents causal interpretation, and self-reported data may be influenced by cultural tendencies toward minimizing distress. While the instruments employed are widely validated, they may not capture the full spectrum of psychological experiences unique to the Somali context. Non-etheless, this research offers foundational evidence in an area previously unexplored in Somalia and underscores the need for future studies examining organizational, cultural, and psychosocial determinants of stress and wellbeing.

Overall, the findings indicate that Somali critical care nurses reported substantial occupational stress alongside moderate levels of psychological wellbeing. The absence of a significant relationship between occupational stress and psychological wellbeing highlights the complexity of this relationship within the Somali critical care context. Strengthening workplace support, improving resources, and implementing mental health interventions will be crucial for sustaining the ICU nursing workforce and enhancing the quality of care within Somalia's evolving healthcare landscape.

### Limitations

This study has several limitations that should be considered when interpreting the findings. The cross-sectional design precludes causal inferences about the relationship between occupational stress and psychological wellbeing. Data were collected via self-report questionnaires administered at a single time point, which may be subject to recall bias, common method variance, and underreporting of psychological distress due to cultural norms. Although validated instruments were used, they may not fully capture culturally specific aspects of stress and psychological wellbeing among Somali nurses. The study was limited to private-sector ICUs. Although all eligible nurses from private hospitals nationwide were included, public-sector nurses were not represented; therefore, the findings are generalizable only to critical care nurses working in private hospitals in Somalia. Future studies should include both healthcare sectors to provide a more comprehensive national perspective. Finally, unmeasured factors, including organizational culture, leadership style, trauma exposure, and social support, may also have influenced psychological wellbeing.

### Recommendation

The findings highlight several practical and policy-related priorities. Strengthening staffing levels and ensuring safe nurse-to-patient ratios should be considered essential steps toward reducing occupational stress in Somali ICUs. Healthcare organizations would benefit from establishing accessible psychological support services, such as counseling programs, peer-support groups, and stress-management training tailored to the demands of critical care practice. Gender-responsive interventions are needed to address the disproportionately lower wellbeing observed among female nurses, including supportive scheduling policies and workplace protections. Policymakers should prioritize regulation of ICU standards in the private sector, expand opportunities for critical care training, and promote retention through improved working conditions and competitive compensation. Future research should explore cultural, organizational, and trauma-related factors influencing stress and resilience among Somali nurses, as well as longitudinal studies to understand how experiences evolve over time. Improving the wellbeing of ICU nurses is essential for strengthening patient safety, service quality, and the long-term stability of Somalia's health workforce.

## Conclusion

This national study provides the first comprehensive picture of how occupational stress and psychological wellbeing interact among critical care nurses working in Somalia's private intensive care units. Although most nurses reported moderate to high stress, their overall psychological wellbeing remained in the mid-range, with relatively strong scores in purpose in life and interpersonal relations. The absence of a significant relationship between occupational stress and psychological wellbeing indicates that this relationship may be influenced by contextual, organizational, or individual factors that were not examined in the present study. Gender differences were notable, with female nurses reporting poorer wellbeing, while nurses with chronic illnesses unexpectedly showed higher wellbeing, indicating potential protective mechanisms that warrant further exploration. Taken together, these findings underscore the need for targeted strategies that prioritize mental health, strengthen workforce infrastructure, and recognize the unique pressures faced by ICU nurses in Somalia. Investing in the wellbeing of this workforce is essential for improving patient outcomes, enhancing retention, and supporting the broader stability of the country's health system.

## Data Availability

The raw data supporting the conclusions of this article will be made available by the authors, without undue reservation.
